# Band Nonlinearity-Enabled Manipulation of Dirac Nodes, Weyl Cones, and Valleytronics with Intense Linearly Polarized Light

**DOI:** 10.1021/acs.nanolett.3c02139

**Published:** 2023-08-14

**Authors:** Ofer Neufeld, Hannes Hübener, Gregor Jotzu, Umberto De Giovannini, Angel Rubio

**Affiliations:** † Center for Free-electron Laser Science, Max Planck Institute for the Structure and Dynamics of Matter, Hamburg 22761, Germany; ‡ Dipartimento di Fisica e Chimica—Emilio Segrè, Università degli Studi di Palermo, Palermo I-90123, Italy; § Center for Computational Quantum Physics (CCQ), The Flatiron Institute, New York, New York 10010, United States

**Keywords:** Nonlinear optics, topological materials, valleytronics, Floquet physics, ARPES

## Abstract

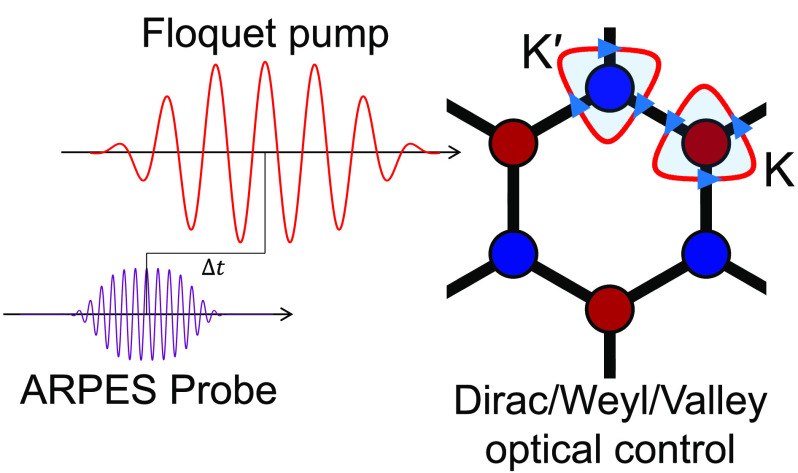

We study low-frequency linearly polarized laser-dressing in materials with valley (graphene and hexagonal-Boron-Nitride) and topological (Dirac- and Weyl-semimetals) properties. In Dirac-like linearly dispersing bands, the laser substantially moves the Dirac nodes away from their original position, and the movement direction can be fully controlled by rotating the laser polarization. We prove that this effect originates from band nonlinearities away from the Dirac nodes. We further demonstrate that this physical mechanism is widely applicable and can move the positions of the valley minima in hexagonal materials to tune valley selectivity, split and move Weyl cones in higher-order Weyl semimetals, and merge Dirac nodes in three-dimensional Dirac semimetals. The model results are validated with ab initio calculations. Our results directly affect efforts for exploring light-dressed electronic structure, suggesting that one can benefit from band nonlinearity for tailoring material properties, and highlight the importance of the full band structure in nonlinear optical phenomena in solids.

Light-induced band structure and optoelectronic device engineering has gained considerable attention in recent years due to its potential to revolutionize electronics.^1−21^ Within this paradigm, a system is irradiated by a coherent laser pulse that dresses the electronic states, potentially changing their properties. The process allows modifying band dispersions, turning insulators into conductors and vice versa, altering the crystal symmetry, and tuning the system’s topology.^2,3,9,15,20−32^

One of the most-studied materials for light-induced band engineering is graphene, which in the absence of driving is a two-dimensional (2D) Dirac semimetal with the band touching at the K and K′ points in the Brillouin zone (BZ). The degeneracies can be lifted when driving the system with circularly polarized light, generating diverse topological phases.^15,18,22,23,26^ The effect is attributed to the breaking of time-reversal symmetry (TRS). The gap opening in light-driven graphene has not yet been observed in angle-resolved photoemission spectroscopy (ARPES) due to various possible experimental limitations,^32−35^ but hybridization gaps have been seen in other systems.^2,36^ On the other hand, topologically trivial gap opening in graphene also occurs without breaking TRS if inversion symmetry is lifted^37,34^ or strain is introduced (moving the Dirac nodes until oppositely charged nodes annihilate to open a gap).^38,39^ It was also shown in optical lattices that by shaking the lattice, one can move the Dirac nodes along high-symmetry axes until they merge,^40,41^ with analogous phenomena occurring in the very intense high frequency driven regimes.^42^

Here we show that with strong-field and low frequency laser driving, a linearly polarized monochromatic field can move the Dirac nodes in the BZ by a substantial amount, and the movement’s direction is fully controlled by the laser polarization. Effectively, this opens a large pseudogap at the original position of the Dirac nodes. We analytically show that the physical mechanism for the effect relies on band nonlinearities and therefore does not appear in the simplest linearized low-energy model of Dirac bands. We validate these results with time-dependent density functional theory (TDDFT) calculations of ARPES spectra. Lastly, we show that this physical mechanism is general and allows versatile control of the band engineering in a wide range of materials. As examples, we demonstrate control over the position of the valley minima and valleytronics in hexagonal-Boron-Nitride (hBN, allowing valleytronics control in transition-metal-dichalcogenides),^43−46^ splitting and moving charge-II Weyl cones,^47^ and merging Dirac nodes in three-dimensional (3D) Dirac semimetals.^48,49^

We begin by analyzing a graphene system with a two-band tight-binding (TB) model with fifth-order nearest-neighbor (NN) terms.^50^ In the basis of creation/annihilation operators on the A/B sublattice sites of the honeycomb lattice, the field-free Hamiltonian is
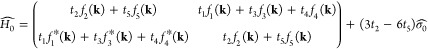
1where  is the identity matrix, *t*_*i*_ are hopping amplitudes to the *i*th NN site, and *f*_*i*_(**k**) are structure factors (see Supporting Information (SI) section I). The second term in eq 1 conveniently sets the top of the valence band to zero energy, while the first term represents the various hopping processes. Note that  inherently does not include , setting the gap to zero and resulting in Dirac cones with local linear dispersion in the K/K′ points. The eigenvalues of , denoted as ϵ_±_(**k**), are obtained analytically. The hopping amplitudes are fitted through least-squares such that ϵ_±_(**k**) match bands obtained from density functional theory (DFT) calculations performed using octopus code^51−53^ within the local density approximation (see SI sections II and III). Notably, the TB model provides very good bands around the K/K′ valleys (the main region of interest) but fails around the Γ-point.

 is coupled to an external laser by Peierls substitution, yielding 
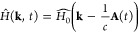
where  is light’s vector potential within the dipole approximation. Here, *E*_0_ is the field amplitude, ω is the driving frequency, *c* is the speed of light, and **ê** is a polarization vector. From this time-periodic Hamiltonian we obtain the Floquet Hamiltonian in the basis of harmonic functions of ω with the sub-blocks:
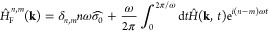
2where |*n* – *m*| is the photon channel order, and the integrals in eq 2 are solved numerically.  is then exactly diagonalized, and the eigen-energies are corrected by their photon-channel index. The resulting Floquet quasi-energy valence and conduction bands, ϵ_±_^*F*^(**k**), are taken as the bands that converge to the field-free bands for *E*_0_ → 0.

Our main interest is the position of the Dirac nodes in the driven system. Since in graphene the Dirac nodes host a nonzero Berry phase,^54−56^ they cannot be removed by a linearly polarized laser field (that does not break inversion or TRS^57^) unless oppositely charged nodes merge.^58^ However, we can still track the nodes’ movements with respect to laser driving. In order to simplify the analysis, we initially ask whether a Floquet quasi-energy gap can open in the original positions of the Dirac nodes at K/K′, defined as *E*_*g*_^*F*^ = ϵ_+_^*F*^(**K**) – ϵ_–_^*F*^(**K**). If a gap opens, the linearly dispersing nodes have moved (note that we will later analyze directly the movement of the nodes). We analyze the Floquet propagator, , and use atomic units unless stated otherwise.  describes time propagation over one laser cycle, and taking the logarithm of its eigenvalues is formally equivalent to diagonalizing the Floquet Hamiltonian.^27^ The propagator can be represented by a time-independent effective Hamiltonian,^26,59^, where  comprises a Magnus series expansion:
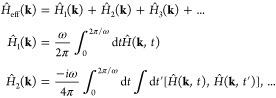
3In this representation  acts as a direct time-averaged Hamiltonian, and higher orders capture effects due to the Hamiltonian not commuting with itself at different times. This notation is especially appealing for analyzing gap openings in graphene, because  does not include  terms; hence,  purely comprises , , and  terms, and  purely comprises gap-opening  terms. The next orders follow such that only even-order terms in the Magnus expansion allow potential gap openings at K/K′.

The main question of interest is under which conditions do the even-order terms vanish. Let us first prove that for the perfectly linear low-energy Dirac Hamiltonian, fields that do not break TRS cannot open a gap at K. For this, we take the first-order expansion of  around K, , where Δ**k** is the momenta away from K and *v*_f_ is the Fermi velocity. Coupling  to an external laser field provides a time-periodic Hamiltonian that is inserted in the Magnus expansion. Due to the linearity of the Dirac Hamiltonian, we obtain  (because ∫_0_^2π/ω^**A**(*t*) = 0). Thus, for the Dirac Hamiltonian, only higher order terms can alter the band structure. For , we find
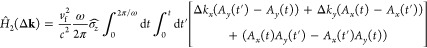
4

There are three main terms inside the integral in eq 4: the first two in the top row vanish at K (Δ**k** = 0). The third term is *k*-independent, and the only one that survives at K. This term clearly vanishes for linear driving since then one of the laser polarization components is zero. We now show that it also vanishes for any TRS field. First, we separate the double integral in the third term in eq 4:
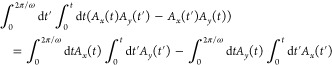
5Next, without loss of generality we represent **A**(*t*) with a pure harmonic sine series, **A**(*t*) = ∑**a**_*m*_ sin (*mωt*), such that the electric field is given by a pure cosine series, **E**(*t*) = , inherently respecting TRS (**E**(*t*) = **E**(−*t*)). Plugging these into eq 5, we note that one polarization component of **A**(*t*) is always integrated over in the *t*′ integral, giving a time-even function, while the other component remains time-odd. The second temporal integral over *t* then vanishes since it integrates a time-odd function. Thus,  in the Dirac Hamiltonian for any TRS drive. In the SI (section VI), we generalize this proof to all even orders of the Magnus expansion. Overall, a Floquet pseudogap cannot open in the low-energy Dirac Hamiltonian driven by a TRS field. This is a well-established result that has been shown with other methodologies. It is, however, potentially misleading because it seemingly pinpoints the physical reason a gap does not open at K to the presence of TRS. Contrarily, we argue that the physical origin of the effect is the linearity of the Hamiltonian (and similarly, Weyl^13^ or other linearly dispersing systems^7^). Indeed, if one repeats the analysis for a field-free parabolic Hamiltonian of the form , the proof no longer holds regardless of TRS. One can verify that in that case, a Floquet gap does open, even though the Hamiltonian is spherically symmetric and in a low-energy continuum form.

For completeness, we repeat the analysis for the TB Hamiltonian at K, keeping only up to second-order NN terms and employing a linearly polarized drive along *k*_*y*_ (respecting TRS).  takes the form:
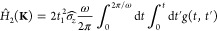
6where the function under the double integral is composed of nested trigonometric functions (see SI section VI) and cannot be analytically integrated. Still, eq 6 can be evaluated numerically, and we have found that generally . The SI (section VI) presents exemplary results for the size of  vs the laser amplitude and wavelength, showing power-law-like scalings. We also note some analytical intuition arises from this analysis, e.g., that the gap at K should scale parabolically with *t*_1_ and be independent of *t*_2_ (as well as *t*_5_) because they only couple to  terms that commute. However, the size of the gap and its scaling with the laser parameters is not expected to correspond well with the size of  because in practical conditions, higher order terms in the magnus expansion cannot be neglected.^60,61^ Moreover, in the fifth-NN TB Hamiltonian, the *t*_1_ hopping term interferes with higher-order terms, leading to more complex dynamics (see SI section VI). Nonetheless, even if not quantitative, this analysis establishes the gap opening at K and its physical origin—if , higher order terms will also be nonzero, and there is no general symmetry constraint that causes their summation to vanish. In the SI (section VI), we provide thorough exact numerical investigations of the size of the pseudogap; it indeed scales parabolically with *t*_1_ and does not scale with *t*_2_, corroborating the analytical analysis. We generally found that the gap at K can be very substantial (up to 0.5 eV). Practically, we recall that this pseudogap means that the Dirac nodes moved elsewhere, where a larger gap suggests the positions have moved further away from K/K′.

Before moving further, it is worth highlighting some noteworthy points: (i) If *E*_0_/ω ∼ 1, the Magnus series can converge very slowly, or even diverge, but it is still valid for determining if a gap opens at K. (ii) The gap at K (and Dirac nodes movement) arises from band nonlinearity in the field-free Hamiltonian away from K, and it vanishes in the limit where the low-energy Dirac Hamiltonian becomes valid. However, simply evaluating the Hamiltonian in the vicinity of K does not guarantee that the low-energy expansion around it is valid; rather, *E*_0_/ω must be sufficiently small. This condition breaks if *E*_0_ is large, or ω is small, as obtained in the strong-field limit, and it is already broken in often-employed conditions for observing Floquet sidebands (powers of ∼10^11^ W/cm^2^ and wavelengths ∼1600 nm open a pseudogap of ∼50 meV and move the Dirac node ∼0.3% of the BZ along *k*_*x*_). (iii) The Dirac node motion strongly depends on the laser orientation, since that greatly changes the Magnus expansion. For instance, for a laser polarized along *k*_*x*_, we obtain *g*(*t*,*t*^′^) = 0, and only even terms beyond the fourth-order in the Magnus expansion are nonzero.

Having established this result, we numerically investigated its dependence on the laser parameters. When graphene is driven along high symmetry axes (along Γ–*M* or Γ–*K*), we find that the Dirac nodes only move along the *k*_*x*_ axis (similarly to shaken optical lattices^40^). Figure 1(a,b) presents the distance of the out-of-equilibrium Dirac nodes from K (Δ*K*) vs laser power and wavelength, which can be quite substantial, and up to ∼10% of the BZ in reasonable experimental conditions. In more extreme cases, oppositely charged Dirac nodes can even merge. We determined that this process requires laser powers of ∼10^13^ W/cm^2^ (at 1600 nm driving wavelength along the *x*-axis), although this value is qualitative because it depends on the details of the TB model around Γ, where it fails. This critical power is slightly higher than graphene’s damage threshold, implying that linearly polarized driving cannot open a proper gap.

**Figure 1:**
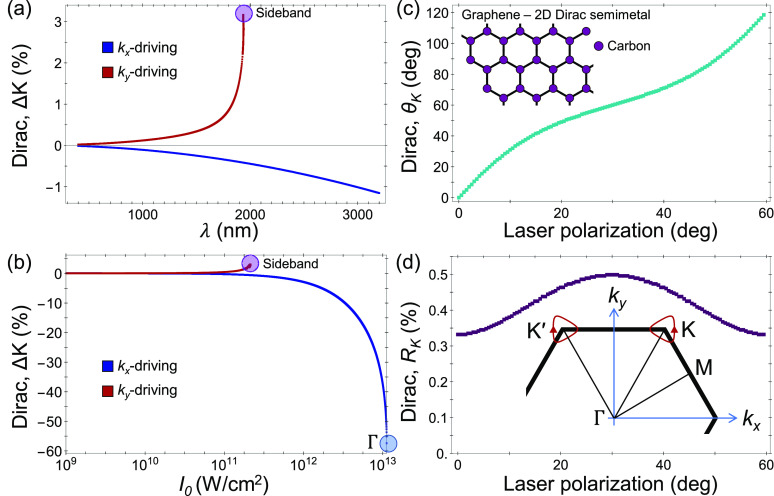
Dirac node motion in graphene driven by linearly polarized light. (a) Floquet–Dirac node distance from the original K-point along the *k*_*x*_-axis vs driving wavelength for a power of 10^11^ W/cm^2^, and driving along *x*- and *y*-axes. (b) Same as (a), but vs driving power for a wavelength of 1600 nm. The highlighted point signifies a Dirac node merger event (blue) and merger events with Floquet replicas (purple). (c) Angle of the Dirac node (with respect to the *k*_*x*_-axis) vs the driving polarization angle (with respect to the *x*-axis), for power of 10^11^ W/cm^2^ and wavelength of 1600 nm. (d) Same as (c), but presenting the radial distance of the Dirac node from its equilibrium position at K. Inset in (d) shows the trajectory (red) of the Dirac nodes around K/K′ in the BZ as the laser polarization rotates (size enhanced for clarity). Inset in (c) shows the graphene lattice.

Slightly different results are observed for *y*-polarized driving, where the Dirac nodes move in the opposite direction (Figure 1(a,b)). Interestingly, here, the Dirac node at K(K′) interacts with the hybridized Floquet sidebands (replicas of K′(K)) until they gradually merge and open a gap for a certain critical pump wavelength and power. At that point, another gapless sideband enters the region. Such dynamics continue for longer wavelengths (or higher intensities), where more sidebands enter the region around K/K′. The effect is similar to phenomena observed in other driving conditions with Dirac point spawning,^26,37^ but seems distinct to very long wavelength driving at which it also becomes difficult to distinguish between Floquet replicas and the original Dirac points (see SI section VI). Thus, measuring the position of the Dirac nodes with respect to the driving parameters could potentially probe additional information about the system such as band hybridization.

Figure 1(c,d) plots the position of the Dirac nodes in the driven system vs the laser polarization axis (both angle, θ_*K*_—the angle of the shifted Dirac point around its original position, and distance, *R*_*K*_—the distances of the shifted Dirac point from its original position; see illustrated trajectories in the inset of Figure 1(d)). As the laser polarization rotates, the Dirac nodes smoothly rotate (with a trigonal pattern) around their equilibrium positions in correspondence. This verifies that a single monochromatic linearly polarized laser can arbitrarily place the Dirac nodes in the BZ.

We next show that these results are not specific to graphene, or even to linearly dispersing systems; band nonlinearity inherently exists in all periodic systems regardless of their low-energy local structure. First, we perform similar calculations in monolayer hBN (see SI section V and ref (62) for details). Figure 2(a,b) shows that the position of the valley minima (defined as the minimal optical gap points in the BZ) moves around with the laser drive and rotates around their equilibrium position by few percent. This provides a potential path to optically tune valley selectivity (also in transition-metal-dichalcogenides) without circular driving, because the local orbital character around the minima point differs from that at K/K′, and the valley minima can be arbitrarily shifted away (whereas with circular driving, it is fixed due to rotational symmetry). This is especially clear if one considers that valley optical selection rules can be explicitly derived only at K/K′ points, while the Bloch states have mixed character in their vicinity.^45,46^ Second, we perform similar calculations in the 3D Dirac semimetal, Na_3_Bi^48,49^ (see SI section V). Crucially, in Na_3_Bi even the low-energy Hamiltonian contains large nonlinearities at the Dirac nodes because they arise from a crossing of two parabolic bands. Figure 2(c) shows that linearly polarized laser driving can move the Dirac nodes just as in graphene. The two nodes merge at laser powers of ∼10^11^ W/cm^2^ (at 1600 nm), which is within experimental feasibility. Physically, this merging is possible in Na_3_Bi because the nodes are initially relatively close to each other. However, this predicted critical power might slightly differ in the realistic system due to the validity of the low energy Hamiltonian around Γ. Third, we calculate the Floquet quasi-energy bands for linearly polarized driven SrSi_2_, which is a Charge-II Weyl semimetal with parabolically dispersing Weyl cones.^47^ Due to the parabolic dispersion, the system is inherently nonlinear (see SI section V). We find that the laser splits the Charge-II Weyl cone into two Charge-I linearly dispersing cones. Figure 2(d) shows that as the driving power increases, the new charge-I cones move further apart. We have found that their motion can be fully controlled within the *xy*-plane, in which the field-free bands are parabolic (following the laser polarization). Along the *k*_*z*_-axis on the other hand, the electronic bands are linearly dispersing and no driving parameters can move the Weyl cones. This highlights that the physical mechanism relies on band nonlinearity.

**Figure 2:**
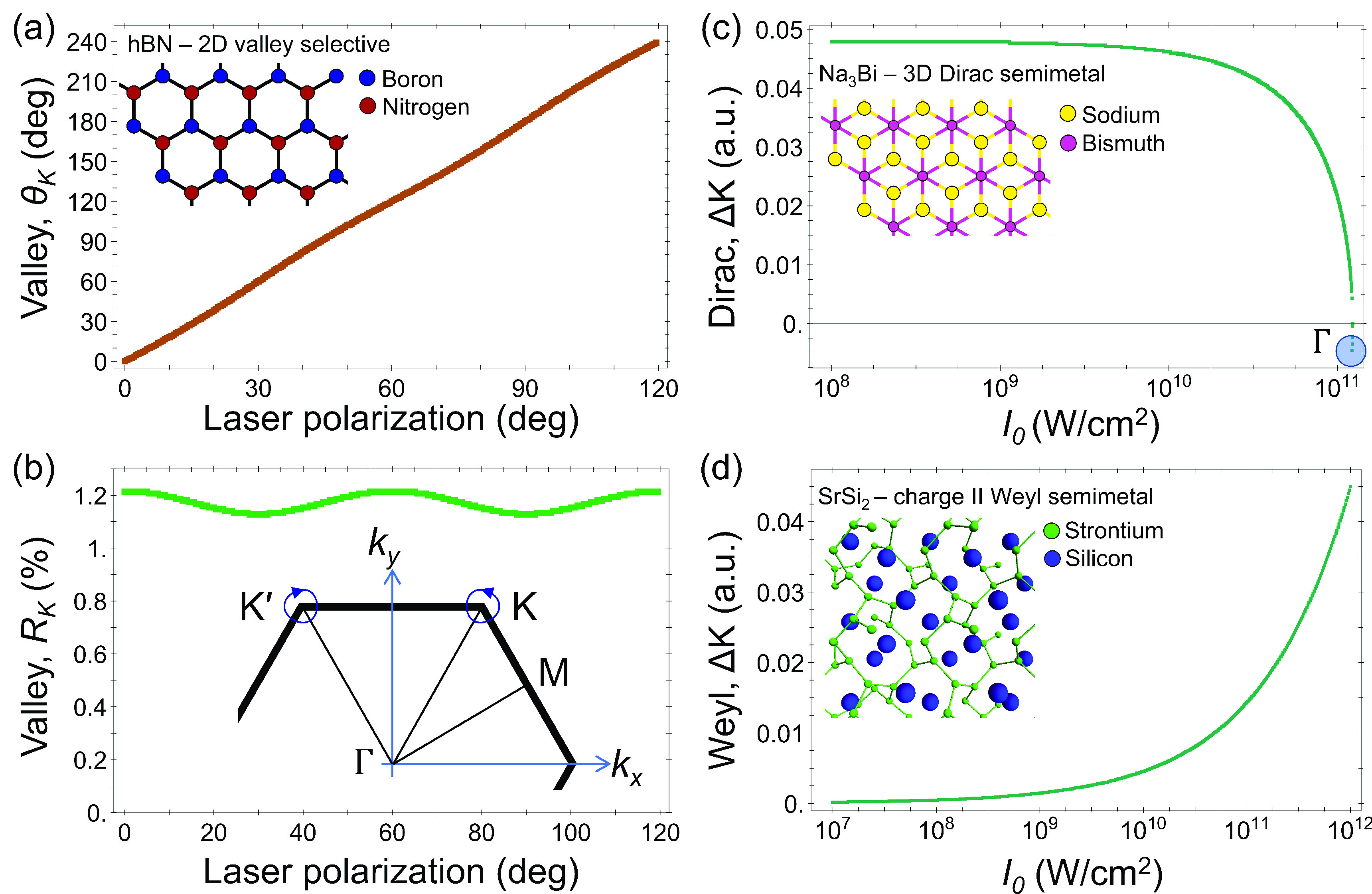
Results in other material systems. (a) Angle of the valley minima point in hBN with the same notations and conditions as those in Figure 1. (b) Same as (a), but presenting the distance of the Dirac node from its equilibrium position at K. (c) Floquet–Dirac node motion in Na_3_Bi—Dirac node distance from the original position vs laser power, for a wavelength of 1600 nm and *x*-axis polarization. The highlighted point signifies a Dirac node merger event. (d) Charge-II Weyl cone splitting, and Charge-I Weyl cone motion, in SrSi_2_—Floquet–Weyl cone distance from its original position vs driving power for similar laser conditions as (c). Inset in (b) shows the trajectory (blue) of the valley minima around K/K′ in the BZ as the laser polarization rotates (size enhanced for clarity). Insets in (a), (c), and (d) show the hBN, Na_3_Bi, and SrSi_2_, lattice structures, respectively.

Lastly, to further establish the model results, we performed ab initio TDDFT calculations of ARPES in light-driven graphene. The methodology follows ref (63), but with artificially doping the conduction band to make the ARPES signals from it more visible. All details of these calculations are delegated to the SI section IV (see also refs (51−53), (64−67)). Figure 3 presents the resulting spectra along *k*_*x*_- and *k*_*y*_-axes overlaid with the quasi-energy bands obtained from the model, which agree remarkably well; a large gap of ∼0.18 eV opens at the original K point (seen when plotting along *k*_*y*_), and the Dirac nodes shifts by ∼1.15% of the BZ along *k*_*x*_. Note that the use of the Dirac Hamiltonian in this case completely fails in describing the spectra because it fixes the Dirac nodes to K/K′. We further emphasize that even though intense pumping is required to observe these phenomena in ARPES, intensities of up to 4 × 10^10^ W/cm^2^ are already achievable,^68^ and work is underway to allow even more intense pumping.^69^ Moreover, by utilizing longer wavelength pumps (e.g., in the THz regime^70^), weaker peak powers can be used to observe similar phenomena (see SI section VI). Regardless, even weaker signals of Dirac point motion could be extracted from experimental spectra by subtracting the field-free backgrounds or utilizing only their asymmetric part. Furthermore, as the motion is polarization-dependent, spectra obtained at different polarizations will help distill the signal. Therefore, these predictions should be experimentally accessible with the current technology.

**Figure 3:**
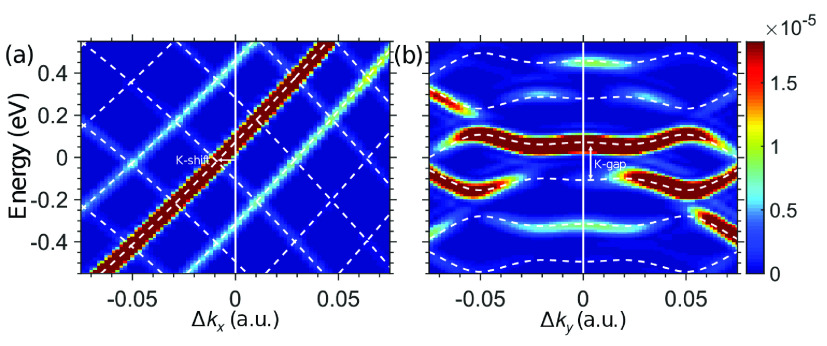
Ab initio TDDFT calculations of ARPES from light-driven graphene for linearly polarized driving along *k*_*x*_ at 3200 nm and 10^11^ W/cm^2^. The spectrum is plotted along *k*_*y*_ (a) and k_*x*_ (b), in the region of K, and is saturated for clarity. The overlaid dashed lines denote the Floquet quasi-energy bands obtained from the model in the same driving conditions. Arrows indicate shifting of the Dirac node and opening a gap at K.

To conclude, we investigated several material systems irradiated by intense low-frequency linearly polarized lasers. For Dirac linearly dispersing systems, we showed that the laser moves the Dirac nodes away from their initial position. This motion is substantial and can be fully controlled by changing the laser parameters (intensity, wavelength, polarization). The effect was analytically shown to originate from band nonlinearities, highlighting the importance of the employed model. Consequently, our results emphasize the obvious yet sometimes overlooked feature that low-energy Hamiltonians fail when driven by sufficiently intense or long-wavelength lasers. We further validated the generality of the physical mechanism with extensive additional calculations, showing that linearly polarized driving can: (i) control the positions of valley minima in valley-selective materials (tuning valleytronics), (ii) merge Dirac nodes in 3D Dirac semimetals, and (iii) split high-order Weyl cones and control the positions of the resulting linearly dispersing cones. We confirmed the model results with ab initio TDDFT calculations and outlined an ARPES setup able to test our predictions.

The present findings should help guide future experiments and theory of Floquet band engineering; and in particular, to benefit from electronic band structure nonlinearities to tailor material properties. Our results also emphasize the importance of the full BZ and band structure away from the minimal gap points in strong-field physics processes in solids, such as high harmonic generation,^71−73^ photogalvanic effects,^74−76^ magneto-optical effects,^77,78^ and more. This is especially relevant in quantum materials and systems with topological or linearly dispersing bands,^70,79−83^ motivating development of ab initio methodologies. We expect that the movement of the high-symmetry points in the BZ will imprint additional characteristics not only directly in ARPES, but also for linear and nonlinear optical responses such as transient absorption spectra and high harmonic generation, which should motivate future work.

## Supporting Information Available

Technical details of the tight-binding model. Technical details about the DFT calculations and fitting procedures of the tight binding hopping amplitudes. Technical details of the TDDFT calculations and ARPES calculations. Technical details of the Floquet calculations in material systems other than graphene. Extended proof that all higher-order even Magnus expansion terms vanish in the Dirac Hamiltonian driven by time-reversal symmetric light. Extended numerical investigation of the pseudogap opening in graphene and its scaling with laser parameters and tight-binding parameters. Additional results of tr-ARPES in graphene for other laser parameters. (PDF)
