# Supplementary material for: Band Nonlinearity-Enabled Manipulation of Dirac Nodes, Weyl Cones, and Valleytronics with Intense Linearly Polarized Light
Source: Nano Lett. 2023 Aug 14;23(16):7568–75. doi: 10.1021/acs.nanolett.3c02139 (PMC10450813; doi:10.1021/acs.nanolett.3c02139)
Supplement: Supplementary file 1 — nl3c02139_si_001.pdf [file nl3c02139_si_001.pdf]

Supplementary information:
Band nonlinearity-enabled manipulation of Dirac nodes, Weyl cones, and valleytronics with intense linearly polarized light

Ofer Neufeld[1,*], Hannes Hübener[1], Gregor Jotzu[1], Umberto De Giovannini[1,2], Angel Rubio[1,3,*]

[1]Max Planck Institute for the Structure and Dynamics of Matter and Center for Free-electron Laser Science, Hamburg 22761, Germany.

[2]Università degli Studi di Palermo, Dipartimento di Fisica e Chimica—Emilio Segrè, Palermo I-90123, Italy.

[3]Center for Computational Quantum Physics (CCQ), The Flatiron Institute, New York 10010, USA.

*Corresponding author E-mails: oneufeld@schmidtsciencefellows.org, angel.rubio@mpsd.mpg.de.

I. GRAPHENE TIGHT-BINDING HAMILTONIAN DETAILS

We provide here additional technical details on the TB Hamiltonian employed throughout the simulations presented in the main text. The lattice primitive vectors are given as $\mathbf{a}_1 = a_0\hat{\mathbf{x}}$, $\mathbf{a}_2 = a_0(-\hat{\mathbf{x}}/2 + \sqrt{3}\hat{\mathbf{y}}/2)$, with the graphene experimental lattice parameter a_0=2.456Å. The NN hopping vectors on this lattice are provided in Table S1 below with the notation $\mathbf{v}_{i,j}$, where i is the order of the hopping process (e.g. i=3 is 3rd NN hopping), and j is the index of the vector (there are either three or six vectors for a given hopping process, see Fig. S1 for illustration). The resulting structure factors $f_i(\mathbf{k})$ are given as:

$$f_m(\mathbf{k}) = \sum_n \exp\{i\mathbf{k}\cdot\mathbf{v}_{m,n}\} \tag{S1}$$

where the sum runs over all existing n's for that particular order of hopping.

Table S1 – NN hopping vectors, $\mathbf{v}_{i,j}$, given in basis of real space vectors in 2D.

NN order	Hopping vectors					
**1**	$a_0\left\{0,\frac{1}{\sqrt{3}}\right\}$	$a_0\left\{-\frac{1}{2},-\frac{1}{2\sqrt{3}}\right\}$	$a_0\left\{\frac{1}{2},-\frac{1}{2\sqrt{3}}\right\}$			
**2**	$a_0\left\{-\frac{1}{2},-\frac{\sqrt{3}}{2}\right\}$	$a_0\left\{\frac{1}{2},\frac{\sqrt{3}}{2}\right\}$	$a_0\{-1,0\}$	$a_0\{1,0\}$	$a_0\left\{-\frac{1}{2},\frac{\sqrt{3}}{2}\right\}$	$a_0\left\{\frac{1}{2},-\frac{\sqrt{3}}{2}\right\}$
**3**	$a_0\left\{1,\frac{1}{\sqrt{3}}\right\}$	$a_0\left\{-1,\frac{1}{\sqrt{3}}\right\}$	$a_0\left\{0,-\frac{2}{\sqrt{3}}\right\}$			
**4**	$a_0\left\{-1,-\frac{2}{\sqrt{3}}\right\}$	$a_0\left\{\frac{1}{2},\frac{5}{2\sqrt{3}}\right\}$	$a_0\left\{-\frac{3}{2},-\frac{1}{2\sqrt{3}}\right\}$	$a_0\left\{\frac{3}{2},-\frac{1}{2\sqrt{3}}\right\}$	$a_0\left\{-\frac{1}{2},\frac{5}{2\sqrt{3}}\right\}$	$a_0\left\{1,-\frac{2}{\sqrt{3}}\right\}$
**5**	$a_0\left\{-\frac{3}{2},-\frac{\sqrt{3}}{2}\right\}$	$a_0\left\{\frac{3}{2},\frac{\sqrt{3}}{2}\right\}$	$a_0\left\{\frac{3}{2},-\frac{\sqrt{3}}{2}\right\}$	$a_0\left\{-\frac{3}{2},\frac{\sqrt{3}}{2}\right\}$	$a_0\{0,\sqrt{3}\}$	$a_0\{0,-\sqrt{3}\}$

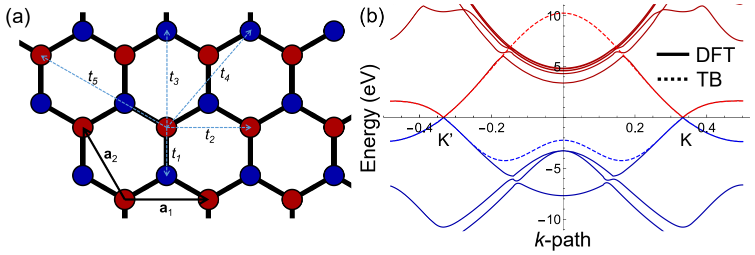

FIG. S1. System illustration. (a) Schematic graphene lattice model with NN hopping terms. Red (blue) denote A (B) sublattice sites, and arrows indicate the different hopping processes and lattice vectors. (b) DFT obtained bands for graphene along a path traversing the BZ from Γ through K in fractional coordinates (solid), compared to the TB model (dashed). Blue and red denote occupied and unoccupied bands, respectively.

II. GROUND-STATE DFT CALCULATIONS

We provide here technical details for performed DFT calculations, which were also employed for obtaining initial states for the TDDFT simulations outlined below (for calculating the ARPES spectra presented in the main text). All DFT calculations were performed with Octopus code[1–3] in a real-space grid representation. The grid was represented on the non-orthogonal primitive unit cell of graphene with equidistant spacings of 0.38 Bohr along the lattice vectors, periodic boundary conditions in the monolayer plane (*xy* plane), and finite boundary conditions along the *z*-axis (where the total length of the *z*-axis was converged at 110 Bohr). We used the experimental lattice parameter of graphene, a_0=2.456Å. A discrete k-grid was converged at a Γ-centered 12x12x1 grid for representing the electron density, but a much finer mesh of 36x36x1 k-points was employed for outputting the band structure for the fitting procedures described below (presented in Fig. S1). Calculations were performed within the local density approximation (LDA) for the exchange-correlation (XC) functional, and while neglecting spin degrees of freedom and spin-orbit coupling. We employed norm-conserving pseudopotentials for describing core states of Carbon[4]. The Kohn-Sham (KS) equations were solved to a strict self-consistency tolerance of 10^{-9} Hartree per unit cell.

III. HOPPING AMPLITUDES FITTING PROCEDURE

From the ground-state DFT calculations we obtained the KS eigenvalues on a finite k-grid, $\epsilon_{KS,n}(\mathbf{k_i})$, where n is the band index and k_i is a grid point in the BZ. The KS valence ($n = 4$) and conduction ($n = 5$) band eigenvalues were then fitted to the TB model bands, $\epsilon_{\pm}(\mathbf{k})$, by employing a least-squares fitting procedure. We optimized the following target function:

$$M(t_1, t_2, t_3, t_4, t_5) = \sum_i \left|\epsilon_{KS,5}(\mathbf{k_i}) - \epsilon_+(\mathbf{k_i})\right| + \sum_i \left|\epsilon_{KS,4}(\mathbf{k_i}) - \epsilon_-(\mathbf{k_i})\right| \qquad \text{(S2)}$$

where the sum included all discrete k-points in the BZ that upheld the condition $\frac{2\pi}{a_0}\left(k_{i,x} + \sqrt{3}k_{i,y}\right) \geq 0.5$. This condition essentially selects points within the K and K' valleys for the fitting procedure (removing points near Γ where the band dispersions invert), and further utilized TRS. The resolution of the k-grid used for fitting was 360x360x1, where the KS eigenvalues for points in-between the original k-grid were linearly-interpolated (with the original grid being 10-fold less dense, 36x36x1). This further guaranteed proper weights were given to the linear region around K and K'. The resulting fitted hopping amplitudes are: -2.0470, 0.4462, -0.0225, 0.1808, and 0.1021 eV, respectively, for t_1-t_5. Comparison between the band structures is presented in Fig. S1.

IV. TDDFT-ARPES CALCULATIONS

We provide here the full details for the *ab-initio* TDDFT-ARPES calculations presented in the main text. We described the laser-induced electron dynamics within the KS-TDDFT framework, where the following KS equations of motion (in atomic units) were solved within the primitive unit-cell of the graphene lattice (with the additional vacuum spacing above and below the monolayer as discussed above):

$$i\partial_t\left|\varphi_{n,k}^{KS}(t)\right\rangle = \left(\frac{1}{2}\left(-i\nabla + \frac{\mathbf{A}(t)}{c}\right)^2 + v_{KS}(\mathbf{r},t)\right)\left|\varphi_{n,k}^{KS}(t)\right\rangle \tag{S3}$$

where $\left|\varphi_{n,k}^{KS}(t)\right\rangle$ is the KS-Bloch state at *k*-point *k* and band index n, $\mathbf{A}(t)$ is the total vector potential of all laser pulses interacting with matter within the dipole approximation, such that $-\partial_t\mathbf{A}(t) = c\mathbf{E}(t)$, *c* is the speed of light in atomic units ($c\approx137.036$). $v_{KS}(\mathbf{r},t)$ in Eq. (S3) is the time-dependent KS potential given by:

$$v_{KS}(\mathbf{r},t) = -\sum_I \frac{Z_I}{|\mathbf{R}_I - \mathbf{r}|} + \int d^3r' \frac{n(\mathbf{r}',t)}{|\mathbf{r}-\mathbf{r}'|} + v_{XC}[n(\mathbf{r},t)] \tag{S4}$$

where Z_I is the charge of the I'th nuclei and $\boldsymbol{R_I}$ is its coordinate (describing the two carbon atoms in the graphene primitive unit cell), v_{XC} is the XC potential that is a functional of $n(\mathbf{r},t)=\sum_{n,k}\left|\left\langle\mathbf{r}\middle|\varphi_{n,k}^{KS}(t)\right\rangle\right|^2$, the time-dependent electron density (where we employed the adiabatic LDA approximation). Note that practically, the bare Coulomb interaction of electrons with the nuclei was replaced with non-local pseudopotentials (described above, assuming the frozen core approximation for core states of Carbon). We neglected coupling to phonons and assumed frozen ions.

These equations were propagated in time from the initial states obtained in ground state DFT calculations (with the 12x12x1 Γ-centered *k*-grid for describing $n(\mathbf{r},t)$), with a time step of 2.9 attoseconds. The main difference here compared to the calculations described above is that the ground state used for the ARPES calculations also involved an artificial doping of the graphene system to populate small electronic charges in the conduction band (making its contribution more visible in ARPES spectra). To this end, we added an additional $0.35\bar{e}$ charge to each unit cell, which was compensated for by an attractive potential arising from the following constructed classical charge density:

$$\rho_{dope}(\mathbf{r}) = N\exp\{-5z^2\} \tag{S5}$$

where $N = 0.02359$ is a normalization constant set such that the total charge from ρ_{dope} integrates to $-0.35\bar{e}$ per unit cell. This guarantees that the system is neutral to avoid issues of charging with periodic boundary conditions. The classical electrostatic potential essentially binds the additional charge on the monolayer, making sure that it occupies graphene bands rather than continuum states. The entire procedure is roughly analogous to the experimental technique of adding a gate potential to dope the conduction band, and only slightly perturbs the overall electronic structure (the band structure is roughly unchanged with and without the doping procedure).

The time-dependent equations of motion were also solved on auxiliary *k*-grids along which the ARPES spectra was calculated. These grids traversed through the K point in the BZ and stretched along k_y, or k_x, with a total of 144 points, starting from $\mathbf{k} = \frac{2\pi}{3a_0}\left(1,\frac{2}{\sqrt{3}}\right)$ and ending at $\mathbf{k} = \frac{2\pi}{3a_0}\left(1,\frac{4}{\sqrt{3}}\right)$ for the grid that is parallel to k_y, and starting at $\mathbf{k} = \frac{2\pi}{a_0}\left(\frac{1}{3}-\frac{\sqrt{3}}{9},\frac{1}{\sqrt{3}}\right)$ and ending at $\mathbf{k} = \frac{2\pi}{a_0}\left(\frac{1}{3}+\frac{\sqrt{3}}{9},\frac{1}{\sqrt{3}}\right)$ for the grid that is parallel to k_x. However, the electron density was not contributed from this grid, but only from the 12x12x1 grid on which the ground state was calculated. During propagation we added a smooth complex absorbing potential (CAP) to avoid spurious reflection of electrons from the boundary. The CAP had a $\sin^2(z)$ shape that saturates to a height of -1 along the *z*-axis grid edges and a total width of 30 Bohr from both sides.

The employed vector potential $\mathbf{A}(t)$, comprised of two pieces – the pump pulse that induces a light-driven Floquet state (discussed in the main text), and an additional probe pulse that photoionizes electrons from the monolayer which can be detected in ARPES experiments. The resulting form is:

$$\mathbf{A}(t) = \mathbf{A}_{\text{pump}}(t) + \mathbf{A}_{\text{probe}}(t) \tag{S6}$$

, with

$$\begin{aligned} \mathbf{A}_{\text{pump}}(t) &= f(t)\frac{cE_0}{\omega}\sin(\omega t)\,\hat{\mathbf{x}} \\ \mathbf{A}_{\text{probe}}(t) &= f_{\text{xuv}}(t-t_0)\frac{cE_{\text{xuv}}}{\omega_{\text{xuv}}}\sin(\omega_{\text{xuv}}t)\hat{\mathbf{z}} \end{aligned} \tag{S7}$$

where $f(t)$ is a temporal envelope function taken to have the following 'super-sine' form[5]:

$$f(t) = \left(sin\left(\pi \frac{t}{T_p}\right)\right)^{\left(\frac{\left|\pi\left(\frac{t}{T_p}-\frac{1}{2}\right)\right|}{\sigma}\right)} \tag{S8}$$

where σ=0.75, T_p is the duration of the laser pulse which was taken to be $T_p=16(2\pi/\omega)$. This form is roughly analogous to a super-gaussian, but where $f(t)$ starts and ends exactly at zero which is numerically convenient. The corresponding full-width-half-max (FWHM) of the pulse is ~85.5 femtoseconds for the chosen ω, which corresponded to 3200nm wavelength driving, assuring the system enters a Floquet steady-state. The envelope function for the probe laser pulse was taken to have a similar form:

$$f_{xuv}(t) = \left(sin\left(\pi \frac{t}{T_{xuv}}\right)\right)^{\left(\frac{\left|\pi\left(\frac{t}{T_{xuv}}-\frac{1}{2}\right)\right|}{\sigma}\right)} \tag{S9}$$

where T_{xuv} is the total duration of the probe pulse taken here as $T_{xuv} = 2000(2\pi/\omega_{xuv})$, which had a FWHM of 51.7 femtoseconds, such that the probe samples multiple cycles of the pump pulse and corresponds to probing Floquet-related physics. The photon energy of the probe was chosen as ω_{xuv}=80 eV, and its intensity was taken as 2×10^8 W/cm^2 to only stimulate single-photon ionization. Both pulses were synchronized to overlap in time such that their peak powers coincided.

The ARPES spectra were calculated directly from the propagated KS states, and without additional fundamental assumptions, using the highly accurate and efficient surface-flux method T-SURFF[6,7]. The momentum-resolved flux of photoelectrons was recorded across a surface normal to the monolayer located at the onset of the CAP. The photoemission from all KS-Bloch states was coherently summed, producing *k*-resolved spectra along the auxiliary *k*-grid. The resulting spectra were smoothed with a moving mean filter and saturated to enhance the visibility of the emission lines.

V. NUMERICAL DETAILS IN OTHER MATERIAL SYSTEMS

1. hBN CALCULATIONS

The TB model Hamiltonian employed for the hBN calculations was equivalent to that in eq. (1) in the main text, but with an added mass term of size Δ (opening a gap of size Δ at K and K')[8], which has the form:

$$\hat{H}_{0,hBN} = \hat{H}_{0,graphene} + \frac{\Delta}{2}(\hat{\sigma_0} + \hat{\sigma_z}) \tag{S10}$$

but where the hopping amplitudes t_i all differ from those chosen from the graphene model. The hopping amplitudes chosen for hBN were fitted to ground-state DFT calculations for its band structure performed with a similar methodology to that described above for graphene (we used the experimental lattice constant of 2.52Å). The resulting employed hopping parameters were -2.1430, 0.3376, -0.0630, 0.1825, and 0.0928 eV, for t_1, t_2, t_3, t_4, and t_5, respectively, and Δ was set at 4.4269 eV (the DFT gap within LDA). The Floquet Hamiltonian and diagonalization procedures were the same as employed in graphene.

2. Na_3Bi CALCULATIONS

The model Hamiltonian employed for the three-dimensional Dirac semimetal Na_3Bi was adapted from the low energy continuum form of the TB expansion in ref.[9]. The resulting 4×4 field-free Hamiltonian has the from:

$$\hat{H}_{0,Na_3Bi} = \epsilon_0(\mathbf{k}) + \begin{pmatrix} M(\mathbf{k}) & A(k_x+ik_y) & & \\ A(k_x-ik_y) & -M(\mathbf{k}) & & \\ & & M(\mathbf{k}) & -A(k_x-ik_y) \\ & & -A(k_x+ik_y) & -M(\mathbf{k}) \end{pmatrix} \tag{S11}$$

where $\epsilon_0(\mathbf{k})= C_0 + C_1{k_z}^2 + C_2(k_x^2+k_y^2)$, $M(\mathbf{k})=M_0 - M_1{k_z}^2 - M_2(k_x^2+k_y^2)$, and all parameter values were taken as in ref.[9]. The Floquet Hamiltonian for the driven system was computed with the same approach used for the 2×2 system calculations, but where the additional z-axis and bands were also considered.

3. $SrSi_2$ CALCULATIONS

The model Hamiltonian employed for the three-dimensional charge-II Weyl semimetal $SrSi_2$ was taken from the low energy expansion in ref.[10] around the Weyl cones, including the spin-orbit interaction terms. The resulting 4×4 field-free Hamiltonian has the from:

$$\hat{H}_{0,SrSi_2} = \begin{pmatrix} v_t k_z & v(k_x - ik_y) & & \\ v(k_x+ik_y) & -v_t k_z & 2\Delta & \\ & 2\Delta & v_t k_z & v(k_x-ik_y) \\ & & v(k_x-ik_y) & -v_t k_z \end{pmatrix} \tag{S12}$$

where for the numerical calculations we employed arbitrary model parameters of v=0.5, v_t=0.7, and Δ=0.125 a.u. The Floquet Hamiltonian for the driven system was computed in the same manner as the other systems.

VI. ADDITIONAL RESULTS IN GRAPHENE

1. DIRAC HAMILTONIAN MAGNUS EXPANSION

In the main text we showed that the 2nd-order term in the Magnus expansion of the Floquet Hamiltonian vanishes for the driven low-energy expanded Dirac Hamiltonian with any field that respects TRS. We present here the generalization of this proof to all higher order even terms. The $2n$'th-order term in the Magnus expansion generally comprises of temporal integrals of commutators of the form:

$$\hat{H}_{2n}(\mathbf{K}) \propto \int dt \int dt' \int dt'' \cdots \left[\hat{H}(\mathbf{k},t), \left[\hat{H}(\mathbf{k},t'), \left[\hat{H}(\mathbf{k},t''), \cdots\right]\right]\right] \tag{S13}$$

where there are $2n$ Hamiltonians appearing taking different time arguments, and $2n$ temporal integrals. All other permutations of the commutators also appear in summation, but it is enough to prove that one of them vanishes because the others are connected by permutations of the temporal arguments. Due to the linearity of the Dirac Hamiltonian, $\hat{H}_D(\mathbf{k})$, and the linearity of the Peierls substitution, the driven Hamiltonians that enter eq. (S10) can be separated into: $\hat{H}(\mathbf{k},t)$=$\hat{H}_D(\mathbf{k})$-$\frac{1}{c}\hat{H}_D(\mathbf{A}(t))$. This results in different terms in the commutators that mix different orders of the vector potential. The terms can be sorted according to their power-law proportionality to the vector potential, ranging from $\propto (E_0)^0$, up to $\propto (E_0)^{2n}$. All of the terms that depend on $\mathbf{k}$ vanish when evaluated at K, and only one term in Eq. (S13) is k-independent, which is the term proportional to $(E_0)^{2n}$. That term arises from substituting in eq. (S13) $\hat{H}(\mathbf{k},t) \to -\frac{1}{c}\hat{H}_D(\mathbf{A}(t))$ for every temporal argument, such that it is necessarily proportional to:

$$\hat{H}_{2n}(\mathbf{K}) \propto \int dt \int dt' \int dt'' \cdots \left[\hat{\boldsymbol{\sigma}}\cdot\mathbf{A}(t), \left[\hat{\boldsymbol{\sigma}}\cdot\mathbf{A}(t'), \left[\hat{\boldsymbol{\sigma}}\cdot\mathbf{A}(t''), \cdots\right]\right]\right] \tag{S14}$$

where here $\hat{\boldsymbol{\sigma}}$ is the vector of Pauli matrices. Since only $\widehat{\sigma_x}$ and $\widehat{\sigma_y}$ terms exist in each $\hat{H}_D$, after $2n$ commutations we are left with one $\widehat{\sigma_z}$ term, which is proportional to:

$$\hat{H}_{2n}(\mathbf{K}) \propto \widehat{\sigma_z} \int dt \int dt' \int dt'' \cdots \sum_{i,j,p} A_i(t)A_j(t')A_p(t'')\cdots \tag{S15}$$

where the indices $i,j,p\ldots$ refer to the cartesian components of the vector potential, which alternate with different permutations for different temporal arguments. Importantly, each of the sums in Eq. (S12) contains a product of $2n$ vector potential terms taking different temporal arguments. Consequently, the first integral can be separated out just like performed in the main text for the 2nd-order term. Another important point is that regardless of the permutations of the cartesian components in the sum in eq. (S12) (which are not described here), every cartesian component of $\mathbf{A}(t)$ upholds TRS on its own. Thus, if the field upholds TRS, then $A_i(t)$ is time-odd for any i, and the first integral generates a pure time-even function. Each subsequent integral flips the parity of the resulting function, because each vector potential in the product takes a unique time argument. After $2n-1$ integrals the resulting function (under the

last integral) is necessarily time-odd, such that the final integral vanishes. This mathematical result occurs in any permutation of the cartesian components and time arguments in the sum in Eq. (S15), and in every permutation of time arguments in eq. (S13). Therefore, we have proven that if the drive respects TRS, all even-order terms in the Magnus expansion of the Floquet Hamiltonian at K vanish for the driven Dirac Hamiltonian, such that there are no gap-opening terms at K. This result relies on the linearity of the Hamiltonian, as discussed in the main text.

2. TB FLOQUET HAMILTONIAN GAP-OPENING TERMS

We explore here the scaling of the first gap-opening term in the Magnus expansion of the Floquet Hamiltonian for the driven TB model of graphene. Specifically, we numerically compute the integrals of the $g(t,t')$ function discussed in the main text for varying laser power and wavelength. The function takes the form:

$$g(t,t') = \sin\left(\frac{S(t,t')}{3}\right)\begin{bmatrix} \cos\left(\frac{S(t,t')}{3}\right) + \cos\left(\frac{4\pi}{3}+\frac{S(t,t')}{3}\right) + \cos\left(\frac{4\pi}{3}-\frac{S(t,t')}{3}\right) \\ -\cos\big(S(t,t')\big) - \cos\left(\frac{2\pi}{3}+S(t,-t')\right) - \cos\left(\frac{2\pi}{3}-S(t,-t')\right) \end{bmatrix} \quad \text{(S16)}$$

with $\mathrm{S}(t,t') = \frac{\sqrt{3}a_0E_0}{4\omega}(\sin(\omega t) - \sin(\omega t'))$. Fig. S2 presents the numerical results showing a cubic power-law scaling with the field amplitude, and 5th power-law scaling with wavelength. Notably, these do not agree with the full numerical results obtained by directly diagonalizing the Floquet Hamiltonian (Fig. S3 in the next SI section). The main reason is that the Magnus expansion converges extremely slowly, and even diverges for high power and long wavelength drives. Nonetheless, the gap term clearly converges to zero in the limit of weak driving, as expected.

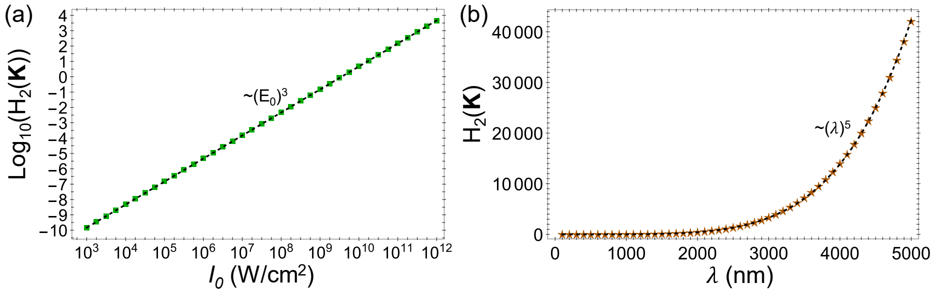

FIG. S2. (a) Scaling of the first gap-opening term in the Magnus expansion of the driven TB model (up to 2nd order TB terms) with wavelength for a driving power of 10^{11} W/cm^2. (b) Same as (a) but with field power and for a wavelength of 1600nm. Dashed black lines present fitted scaling laws.

We also present here the analytic expression for the 2nd-order term in the Magnus expansion of the driven 5th-order TB Hamiltonian (where the main text and Fig. S2 only discuss the driven 2nd-order NN TB model while setting $t_3 = t_4 = t_5 = 0$). The resulting $\hat{H}_2(\mathbf{k})$ for the same condition explored in the main text (k_y driving by a linearly-polarized field) is:

$$\hat{H}_2(\mathbf{K}) = i\hat{\sigma}_z\frac{\omega}{\pi}\int_0^{\frac{2\pi}{\omega}} dt \int_0^t dt' h(t,t') \quad \text{(S17)}$$

where the function under the integral is:

$$h(t,t') = \sin\left(\frac{\kappa\sin\omega t}{2}\right)\sin\left(\frac{\kappa\sin\omega t'}{2}\right)\begin{bmatrix} e^{i\kappa\frac{\sin\omega t-\sin\omega t'}{6}}p(t)p^*(t') \\ -e^{i\kappa\frac{\sin\omega t'-\sin\omega t}{6}}p^*(t)p(t') \end{bmatrix} \quad \text{(S18)}$$

where

$$p(t) = t_4 - t_1 + t_3 - i(t_3 - 2t_4)\sin(\kappa\sin\omega t) + t_3\cos(\kappa\sin\omega t) \quad \text{(S19)}$$

, and with $\kappa = \frac{\sqrt{3}a_0E_0}{2\omega}$. Numerical integration of eq. (S17) leads to very similar results to those presented in Fig. S2 for the 2nd-order TB model (not presented), but the main difference here is that the interference between the different

hopping amplitudes becomes apparent. In particular, different hopping terms contribute to separate parts of the integrand in eq. (S17). This further highlights the role of the full band structure in determining the gap opening at K.

3. K-GAP-OPENING

We numerically investigate here the size of the gap opening in graphene at K *vs.* the laser driving parameters, which complements the analysis presented in the main text for the position of the Dirac nodes in the BZ. Figure S3 shows the resulting exemplary Floquet gaps at K for various conditions, and their scaling with: (a) the NN hopping amplitude t_1, (b) the next-NN hopping amplitude t_2, (c) driving wavelength, and (d) driving power, for the case of a laser polarized along k_y. The gap indeed scales parabolically with t_1, and is independent of t_2, as expected from the analytical analysis. It exhibits an initial parabolic power-law scaling with the driving field amplitude (up until deviations appear from ~5×10^{10} W/cm^2 onwards), and an initial parabolic scaling with wavelength (until deviations arise from ~1000nm onwards). We also note that due to higher order NN hopping terms (that can have opposite signs), there can be non-trivial interference terms that cause gap closing and re-opening (e.g. *vs.* wavelength in Fig. S3(c) at ~2500nm, or *vs.* power in Fig. S3(d) at ~6×10^{11} W/cm^2). This arises when the vector potential term increases in magnitude and probes different regions in the bands (e.g. where their dispersion flips), and connects with the oscillations of the Dirac node around K discussed in the main text for y-polarized driving.

Figures S3(e) and (f) present the gap scaling with laser wavelength and power when the drive is polarized along k_x, which leads to slightly different behavior, but a similar initial scaling with field power and wavelength. The differences arise due to the distinct shape of the field-free bands along those lines. In particular, k_x driving tends to open a larger gap that can be as high as ~0.5 eV in strong-field driving conditions. Note that in principle, such gap opening and re-closing dynamics could also be approximately described by effective time-independent Hamiltonians with the ground-state tunneling amplitudes modified by the laser (e.g. as in ref.[11]).

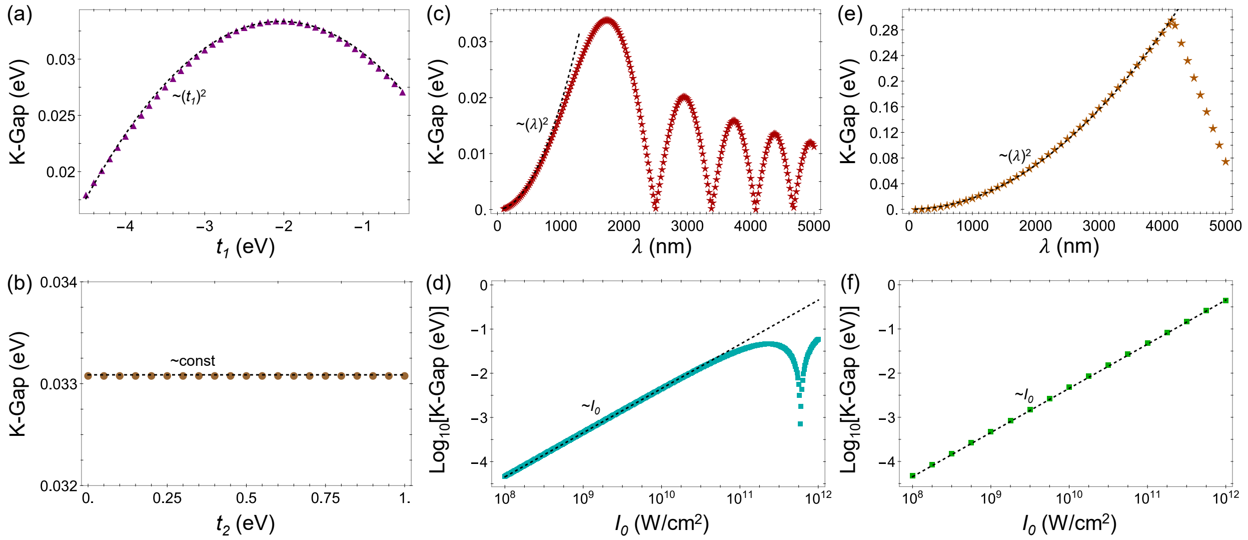

FIG. S3. Analysis of pseudo-gap opening in graphene driven by linearly-polarized light. (a) Floquet gap at K calculated numerically with the TB model for a laser power of 10^{11} W/cm^2 and 1600nm wavelength polarized along k_y *vs.* the NN hopping amplitude, t_1. (b) Same as (a), but *vs.* 2nd NN hopping amplitude, t_2. (c) Same as (a) but for changing wavelength. (d) Same as (a) but for changing power, where the gap is shown in log scale. (e,f) Same as (c,d), but for driving along the k_x axis. Dashed black lines in all cases present fitted scaling laws as discussed in the main text.

4. Interactions with Floquet replicas

We numerically explore the Floquet band structure *vs.* the pumping wavelength in the strong-field regime, and specifically, the Dirac point merger event with nearby sidebands indicated in Fig. 1(a) in the main text. Similar events occur when increasing the driving power as well. Fig. S4 shows the Floquet band structure while tuning the driving wavelength. The original Dirac point (which starts at K in the field-free case, and high frequency regime) slightly shifts right along the k_x-axis as the wavelength increases, until another sideband replica of K' approaches from the

right. For a critical wavelength of ~1940 nm the two points merge. At this stage another gapless sideband approach from the left.

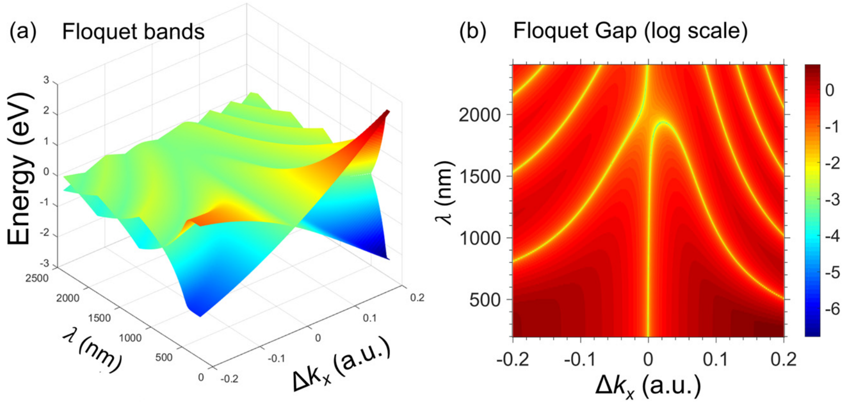

FIG. S4. Floquet band structure and Floquet quasi-energy gap along the k_x-axis for driving conditions similar to Fig. 1(a) in the main text along the y-axis, and *vs.* the driving wavelength. (a) Conduction and valence bands *vs.* driving wavelength, showing the motion of the Dirac point as the drive wavelength is tuned, up until it merges with a nearby replica band. (b) Same as (a), but showing the Floquet quasi-energy gap in logarithmic scale. Plot calculated with the same methodology as in Fig. 1 in the main text.

5. LONG WAVELENGTH ARPES

We numerically explore here ARPES signals from the Floquet pumped system with TDDFT, as in Fig. 3 in the main text, but in the case of longer wavelength driving, and weaker pulse peak power. Figure S5 shows ARPES spectra analogous with Fig. 3(a) in the main text, but for pumping wavelength of 4500nm, and peak power of 4×10^{10} W/cm^2 (which is achievable with current technology[12]). The spectra clearly show that in the long wavelength pumping regime (since the vector potential is large), large movements of the Dirac point in graphene can still be observed even in lower peak powers.

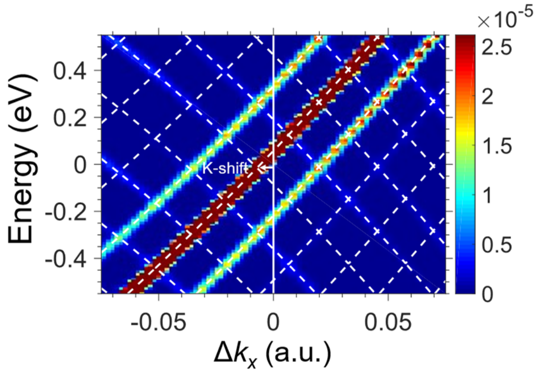

FIG. S5. *Ab-initio* TDDFT calculations of ARPES from light-driven graphene for linearly-polarized driving along k_x at 4500nm and 4×10^{10} W/cm^2. The spectrum is plotted along k_x in the region of K, and is saturated for clarity. The overlayed dashed lines denote the Floquet quasi-energy bands obtained from the model in the same driving conditions. Arrows indicate shifting of the Dirac node and opening a gap at K.

SUPPLEMENTARY REFERENCES

(1) Castro, A.; Appel, H.; Oliveira, M.; Rozzi, C. A.; Andrade, X.; Lorenzen, F.; Marques, M. A. L.; Gross, E. K. U.; Rubio, A. Octopus: A Tool for the Application of Time-Dependent Density Functional Theory. *Phys. status solidi* **2006**, *243* (11), 2465–2488. https://doi.org/10.1002/pssb.200642067.

(2) Andrade, X.; Strubbe, D.; Giovannini, U. De; Larsen, H.; Oliveira, M. J. T.; Alberdi-rodriguez, J.; Varas, A.; Theophilou, I.; Helbig, N.; Verstraete, M. J.; Stella, L.; Nogueira, F.; Castro, A.; Marques, M. A. L.; Rubio, A. Real-Space Grids and the Octopus Code as Tools for the Development of New Simulation Approaches for Electronic Systems. *Phys. Chem. Chem. Phys.* **2015**, *17*, 31371–31396. https://doi.org/10.1039/C5CP00351B.

(3) Tancogne-Dejean, N.; Oliveira, M. J. T.; Andrade, X.; Appel, H.; Borca, C. H.; Le Breton, G.; Buchholz, F.; Castro, A.; Corni, S.; Correa, A. A.; De Giovannini, U.; Delgado, A.; Eich, F. G.; Flick, J.; Gil, G.; Gomez, A.; Helbig, N.; Hübener, H.; Jestädt, R.; Jornet-Somoza, J.; Larsen, A. H.; Lebedeva, I. V; Lüders, M.; Marques, M. A. L.; Ohlmann, S. T.; Pipolo,

S.; Rampp, M.; Rozzi, C. A.; Strubbe, D. A.; Sato, S. A.; Schäfer, C.; Theophilou, I.; Welden, A.; Rubio, A. Octopus, a Computational Framework for Exploring Light-Driven Phenomena and Quantum Dynamics in Extended and Finite Systems. *J. Chem. Phys.* **2020**, *152* (12), 124119. https://doi.org/10.1063/1.5142502.

(4) Hartwigsen, C.; Goedecker, S.; Hutter, J. Relativistic Separable Dual-Space Gaussian Pseudopotentials from H to Rn. *Phys. Rev. B* **1998**, *58* (7), 3641–3662. https://doi.org/10.1103/PhysRevB.58.3641.

(5) Neufeld, O.; Cohen, O. Background-Free Measurement of Ring Currents by Symmetry-Breaking High-Harmonic Spectroscopy. *Phys. Rev. Lett.* **2019**, *123* (10), 103202. https://doi.org/10.1103/PhysRevLett.123.103202.

(6) Scrinzi, A. T-SURFF: Fully Differential Two-Electron Photo-Emission Spectra. *New J. Phys.* **2012**, *14* (8), 085008. https://doi.org/10.1088/1367-2630/14/8/085008.

(7) De Giovannini, U.; Hübener, H.; Rubio, A. A First-Principles Time-Dependent Density Functional Theory Framework for Spin and Time-Resolved Angular-Resolved Photoelectron Spectroscopy in Periodic Systems. *J. Chem. Theory Comput.* **2017**, *13* (1), 265–273. https://doi.org/10.1021/acs.jctc.6b00897.

(8) Galler, A.; Rubio, A.; Neufeld, O. Mapping Light-Dressed Floquet Bands by Highly Nonlinear Optical Excitations and Valley Polarization. arXiv (Optics), 27/03/2023, https://arxiv.org/abs/2303.15055. (Accessed 2023-07-28).

(9) Wang, Z.; Sun, Y.; Chen, X.-Q.; Franchini, C.; Xu, G.; Weng, H.; Dai, X.; Fang, Z. Dirac Semimetal and Topological Phase Transitions in A3Bi (A=Na,K,Rb). *Phys. Rev. B* **2012**, *85* (19), 195320. https://doi.org/10.1103/PhysRevB.85.195320.

(10) Huang, S.-M.; Xu, S.-Y.; Belopolski, I.; Lee, C.-C.; Chang, G.; Chang, T.-R.; Wang, B.; Alidoust, N.; Bian, G.; Neupane, M.; Sanchez, D.; Zheng, H.; Jeng, H.-T.; Bansil, A.; Neupert, T.; Lin, H.; Hasan, M. Z. New Type of Weyl Semimetal with Quadratic Double Weyl Fermions. *Proc. Natl. Acad. Sci.* **2016**, *113* (5), 1180–1185. https://doi.org/10.1073/pnas.1514581113.

(11) Jotzu, G.; Messer, M.; Desbuquois, R.; Lebrat, M.; Uehlinger, T.; Greif, D.; Esslinger, T. Experimental Realization of the Topological Haldane Model with Ultracold Fermions. *Nature* **2014**, *515* (7526), 237–240. https://doi.org/10.1038/nature13915.

(12) Dong, S.; Beaulieu, S.; Selig, M.; Rosenzweig, P.; Christiansen, D.; Pincelli, T.; Dendzik, M.; Ziegler, J. D.; Maklar, J.; Xian, R. P.; Neef, A.; Mohammed, A.; Schulz, A.; Stadler, M.; Jetter, M.; Michler, P.; Taniguchi, T.; Watanabe, K.; Takagi, H.; Starke, U.; Chernikov, A.; Wolf, M.; Nakamura, H.; Knorr, A.; Rettig, L.; Ernstorfer, R. Observation of Ultrafast Interfacial Meitner-Auger Energy Transfer in a van Der Waals Heterostructure. arXiv (Materials Science), 15/08/2021, https://arxiv.org/abs/2108.06803. (Accessed 2023-07-28).
